# Supplementary material for: Growth Inhibition and Additive Effect to Antimalarial Drugs of Brucea javanica Extracts on Asexual Blood-Stage Plasmodium falciparum
Source: Pathogens. 2025 Jun 30;14(7):646. doi: 10.3390/pathogens14070646 (PMC12301036; doi:10.3390/pathogens14070646)
Supplement: Supplementary file 1 [file pathogens-14-00646-s001.zip › pathogens-3697506-supplementary.pdf]

Supplementary data

Method S1: Investigation of RBC toxicity of plant extracts by hemolysis assay.

A 200 mg/mL stock solution of *B. javanica* crude extracts was prepared by dissolving it in DMSO (Merck, United States) and then diluting it with sterile distilled water to a 10 mg/mL working solution. The O-type RBCs in 1x PBS (5% hematocrit) were tested with the plant extracts at increasing doses (0.01 μg/mL to 1,000 μg/mL) in 96-well plates, treated for 0, 1, 6, 18, and 24 hours at 37 °C. The positive control was a 0.1% Triton X solution, and 0.5% DMSO in 1x PBS was used as the negative control. All samples were performed in duplicate in each dilution and were repeated three times. Each sample was measured at an absorbance wavelength of 540 nm using a microplate reader (Tecan Spark 10M, Switzerland). The percentage of hemolysis was calculated and compared with the positive and negative controls. The significance level was considered as $p \leq 0.05$. The result is as shown in Fig. S1.

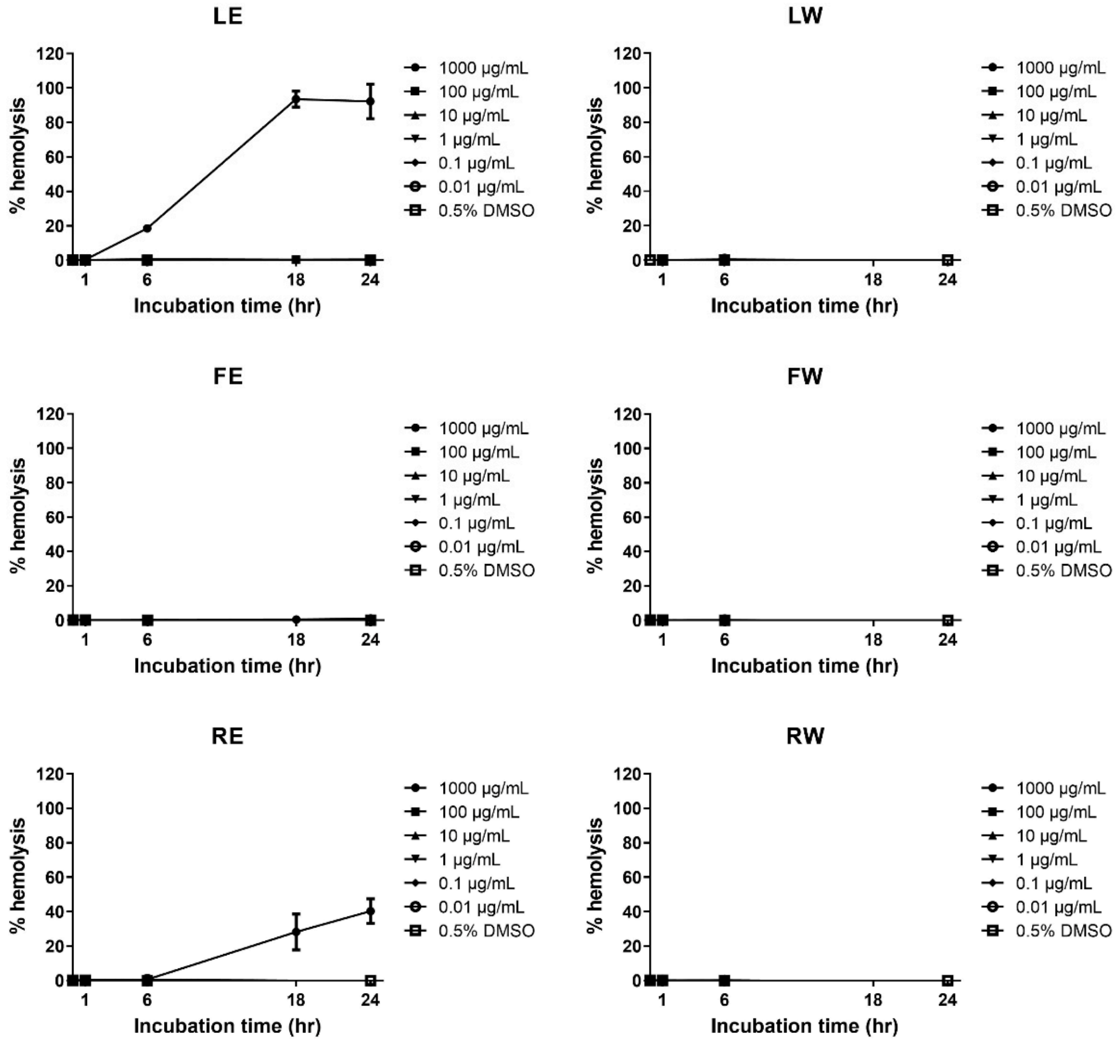

Figure S1. Investigation of RBC toxicity of plant extracts by hemolysis assay. The representative results of the toxicity testing of all parts of *B. javanica* crude extracts on human RBCs by *in vitro* hemolysis assay. The crude extracts from leaves, fruits, and roots, obtained using ethyl acetate, were labeled as LE, FE, and RE, and the crude extracts from leaves, fruits, and roots, obtained by water, were designated as LW, FW, and RW, respectively.

Method S2: Investigation of growth inhibition

The infected RBC samples from the growth inhibition assay were collected to examine the parasitemia by Giemsa-stained blood smear and quantify the amount of *P. falciparum* by real-time RT-PCR. For the Real-time RT-PCR, total RNA was extracted from the infected RBC samples using Trizol Reagent (Life Technologies; United States), followed by the RNA GF-1 entire RNA extraction kit (Vivantis; Malaysia). The RNA was reverse-transcribed to cDNA using ReverTra Ace® qPCR RT MATSer Mix with gDNA remover (TOYOBO; Japan). A real-time RT-PCR assay was performed using iTaq Universal SYBR Green Supermix (Bio-Rad; United States). The copy number of *P. falciparum* 18S small subunit ribosomal RNA (*Pf*18SrRNA) was performed as the target gene, containing a 352-base pair. The forward primer was 5′GCTCTTTCTTGATTTCTTGGATGGT3′ and the reverse primer was 5′CTAGGACATCACAGACCTGTTGTTGC3′. The reaction mixture was incubated at 95°C for 5 minutes, followed by 40 cycles of 95°C for 15 seconds, 60°C for 30 seconds, and 72 °C for 1 minute. The relative copy number of *Pf*18SrRNA in the infected RBC samples was estimated from a standard curve created by plotting the log initial copy number of the control plasmid. The percentage of growth inhibition of all plant extracts, as determined by thin blood smears and Real-time RT-PCR investigations, was calculated and compared with the positive controls. The statistical differences were demonstrated with $p \leq 0.05$. The result is as shown in Fig. S2.

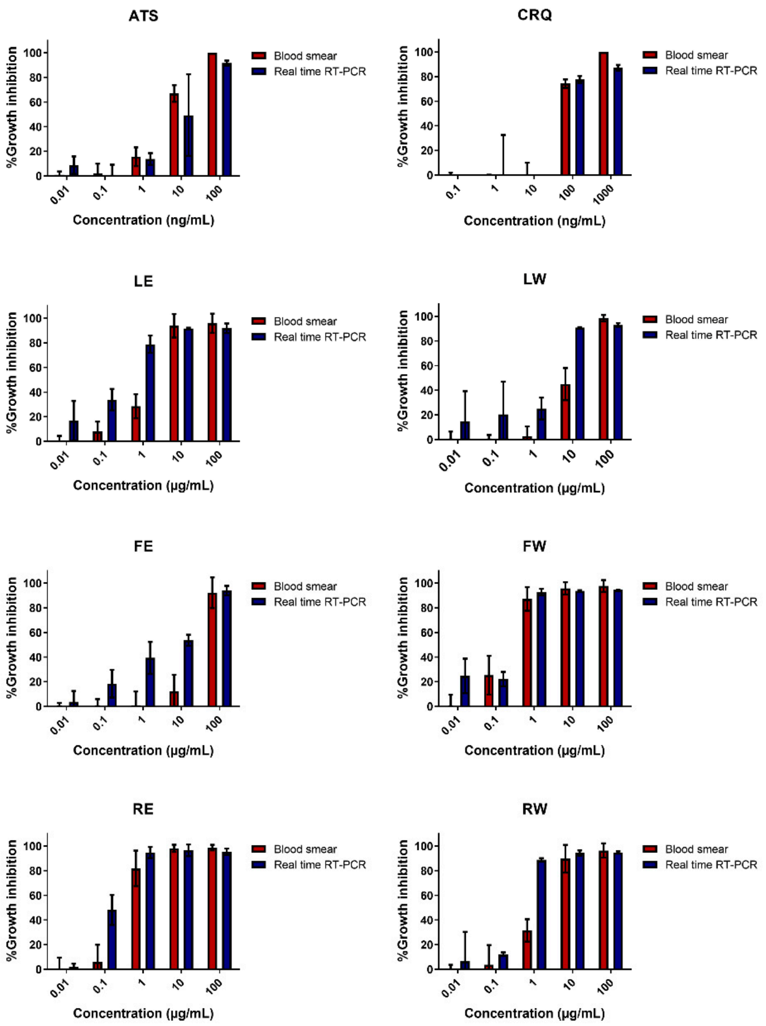

Figure S2. **Percentage of growth inhibition compared between the microscopic technique and Real-time RT-PCR.** The crude extracts from leaves, fruits, and roots, obtained using ethyl acetate, were labeled as LE, FE, and RE, and the crude extracts from leaves, fruits, and roots, obtained by water, were designated as LW, FW, and RW, respectively.

Method S3: Assessment of the reversibility of antimalarial activity of *Brucea javanica* extracts

Synchronized *P. falciparum* ring-stage parasites were treated with different concentrations (100, 10, 1, and 0.1 µg/mL) of *B. javanica* root extract (RE) and fruit extract (FW) for 24-26 hours. After treatment, the culture medium was replaced with fresh medium without extract, and the cultures were continued for an additional 24 hours. Parasite development was monitored by Giemsa-stained thin smears. At high concentrations (100 and 10 µg/mL), parasites treated with RE and FW remained arrested at the ring stage after drug removal, indicating irreversible inhibition of parasite development. At lower concentrations (1 and 0.1 µg/mL), partial recovery of parasite growth was observed. Data were presented as mean ± SD from independent experiments. The result is as shown in Fig. S3.

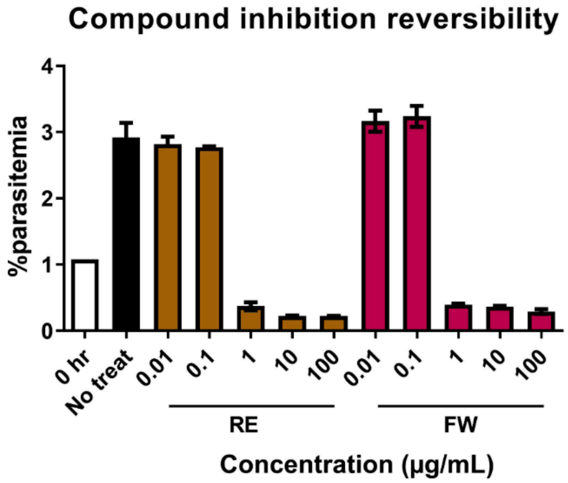

Figure S3. **Irreversible inhibition of *Brucea javanica* extracts on malaria parasite development.** RE represents *B. javanica* root extract extracted by ethyl-acetate, and FW represents *B. javanica* fruit extracted by water.
